# Protein encoding genes in an ancient plant: analysis of codon usage, retained genes and splice sites in a moss, *Physcomitrella patens*

**DOI:** 10.1186/1471-2164-6-43

**Published:** 2005-03-22

**Authors:** Stefan A Rensing, Dana Fritzowsky, Daniel Lang, Ralf Reski

**Affiliations:** 1 Plant Biotechnology, Faculty of Biology, University of Freiburg, Schaenzlestr. 1, 79104 Freiburg, Germany

## Abstract

**Background:**

The moss *Physcomitrella patens *is an emerging plant model system due to its high rate of homologous recombination, haploidy, simple body plan, physiological properties as well as phylogenetic position. Available EST data was clustered and assembled, and provided the basis for a genome-wide analysis of protein encoding genes.

**Results:**

We have clustered and assembled *Physcomitrella patens *EST and CDS data in order to represent the transcriptome of this non-seed plant. Clustering of the publicly available data and subsequent prediction resulted in a total of 19,081 non-redundant ORF. Of these putative transcripts, approximately 30% have a homolog in both rice and *Arabidopsis *transcriptome.

More than 130 transcripts are not present in seed plants but can be found in other kingdoms. These potential "retained genes" might have been lost during seed plant evolution. Functional annotation of these genes reveals unequal distribution among taxonomic groups and intriguing putative functions such as cytotoxicity and nucleic acid repair.

Whereas introns in the moss are larger on average than in the seed plant *Arabidopsis thaliana*, position and amount of introns are approximately the same. Contrary to *Arabidopsis*, where CDS contain on average 44% G/C, in *Physcomitrella *the average G/C content is 50%. Interestingly, moss orthologs of *Arabidopsis *genes show a significant drift of codon fraction usage, towards the seed plant. While averaged codon bias is the same in *Physcomitrella *and *Arabidopsis*, the distribution pattern is different, with 15% of moss genes being unbiased.

Species-specific, sensitive and selective splice site prediction for *Physcomitrella *has been developed using a dataset of 368 donor and acceptor sites, utilizing a support vector machine. The prediction accuracy is better than those achieved with tools trained on *Arabidopsis *data.

**Conclusion:**

Analysis of the moss transcriptome displays differences in gene structure, codon and splice site usage in comparison with the seed plant *Arabidopsis*. Putative retained genes exhibit possible functions that might explain the peculiar physiological properties of mosses.

Both the transcriptome representation (including a BLAST and retrieval service) and splice site prediction have been made available on , setting the basis for assembly and annotation of the *Physcomitrella *genome, of which draft shotgun sequences will become available in 2005.

## Background

Flowering plants have developed from a common ancestor with mosses, liverworts, ferns, and gymnosperms over the last 450 million years [1]. Most recent angiosperms do not closely resemble their ancestors, as known from the fossil record. Quite a few gymnosperms (like *Ginkgo *or *Cycas*) still resemble the plants known from the fossil record, and this is even more true for "lower" land plants, namely mosses, liverworts and ferns [2,3]. In addition, mosses seem to evolve with a slow molecular clock [4]. So, if these plants appear to be more ancient than modern flowering plants as measured by morphological means and mutation rate, does this also hold true for how they employ their genetic system?

A lot of comparative studies on protein encoding genes have already been carried out within and between the two major groups of flowering plants, mono- and dicotyledons, with rice (*Oryza sativa*) and *Arabidopsis thaliana *as the most prominent examples. Currently more than two million *Liliopsida *(monocotyledons) EST are publicly available, the corresponding number for the *Magnoliophyta *(dicotyledons) even exceeds four million sequences. However, sequence information for other plant phyla is still scarce. There are only about 160,000 EST sequences available of both *Coniferophyta *(part of the gymnosperms) and *Chlorophyta *(green algae), 130,000 from *Bryophyta *(mosses) and 3,700 from *Filicophyta *(ferns) (all numbers from Genbank). For the moss *Physcomitrella patens*, more than 102,000 nucleic acid sequences (mainly EST) are publicly available to date. This "ancient" land plant therefore is an ideal candidate to unravel some details about how simple plants encode proteins and whether they do so in a different manner from "modern" plants, as represented by the monocotyledon rice and the dicotyledon *Arabidopsis *in this study.

*Physcomitrella *is increasingly being used as a model plant because of its unrivalled capability among plants to include ectopic DNA into its genome by means of homologous recombination (see e.g. [5-7]), thus enabling gene replacement in a straight forward manner. As in all mosses, the haploid gametophyte is the dominant generation in the heteromorphic life cycle. In this respect the moss is different from seed plants (gymnosperms and flowering plants), in which the polyploid sporophyte dominates the life cycle. It has been argued before [8,9] that the set of genes of the respective dominant generation is equivalent, while a large proportion of moss transcripts cannot currently be assigned a putative function. These "orphan" genes might encode functions that are specific to mosses and are not present in other taxonomic groups. Besides species-specific orphan genes, mosses might also possess retained genes, that have been lost in seed plants during evolution. Both types of genes are candidates to encode functions that make mosses unique in terms of physiology and metabolism. For example, *Physcomitrella *exhibits increased tolerance towards abiotic stresses [10,11], uses proteins derived from the same gene in different cellular compartments by dual targeting [12,13] and displays secondary metabolite pathways not known in seed plants [14-16]. In this study, following up on the initial analyses by Nishiyama et al. [8], we aimed to increase our knowledge of the moss transcriptome.

## Results and discussion

### Comparative BLAST searches

**Figure 1:**
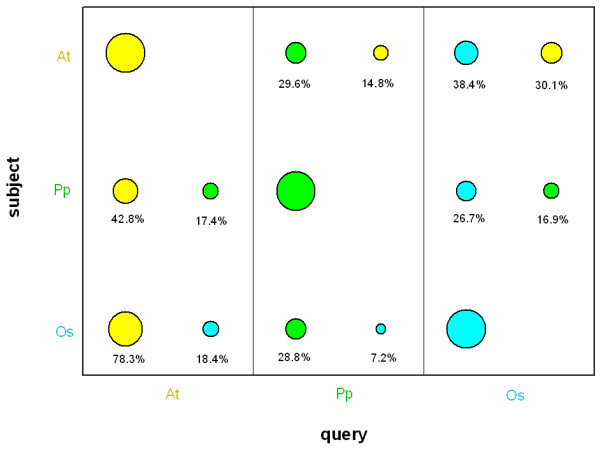
**Comparative BLAST searches between *Arabidopsis*, rice and moss**. Comparative BLAST searches of the *Arabidopsis *(At, yellow), rice (Os, cyan) and *Physcomitrella *(Pp, green) transcriptomes. Each search was done with the respective sets once as query and once as search space (subject). The area of the circles represents the percentage of the query/subject sequence space that yielded filtered hits.

**Figure 2:**
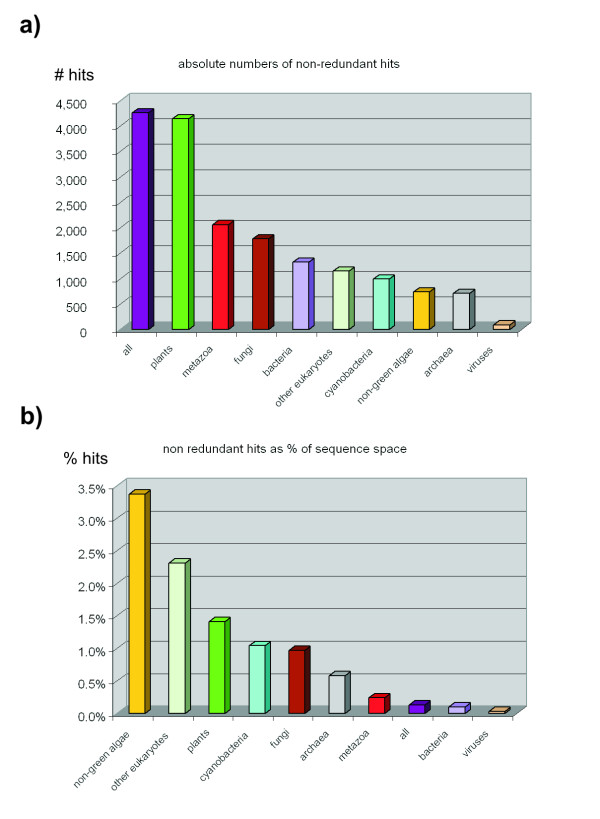
**BLAST hits of *Physcomitrella *protein genes against the taxprot dataset**. a) Absolute number of hits against different taxonomic groups. b) Amount of non-redundant hits as percentage of the respective sequence space.

Around 30% of the *Physcomitrella *ORF have homologs in both rice and *Arabidopsis *transcriptome whereas 80% of the *Arabidopsis *genes have a homolog in rice and 40% of the rice genes in *Arabidopsis *(Fig. 1). Although these numbers are lower than the actual amount of sequence homologs because of filtering (see below), they demonstrate that *Physcomitrella *contains a lot of as yet unknown protein encoding genes that might be specific for mosses. A homology search against the taxprot dataset (Table 1, Fig. 2) reveals that 45.8% of the predicted moss ORF find a query in plants (E-value threshold 1E-4), after rigorous filtering 28.1% remain and 21.7% are non-redundant, i.e. do not match multiple subject sequences. The rigorous filtering (see methods for details) for true homologs thus necessarily decreases the set of available sequences, so that false conclusions are not made based on comparison of non-homologous sequences.

**Table 1:** Taxonomic constitution of the taxprot dataset

**taxonomic group**	**txid**	**# of sequences**^1^
Metazoa	33208	862,420
Fungi	4751	184,282
Viridiplantae (plants and green algae)	33090	293,156
Non-green algae^2^		21,889
Other Eukaryotes^3^		49,732
Eubacteria (without Cyanobacteria)	2	1,386,089
Cyanobacteria	1117	94,920
Archaea	2157	122,394
Viruses	10239	331,246
**Total**		**3,346,128**

^1^Genbank amino acid sequences as of 2004–04–07, NCBI taxon ids are shown under "txid", all taxonomic crown groups with at least 100 sequence members were used; ^2^Cercozoa [136419], Cryptophyta [3027], Euglenozoa [33682], Glaucocystophyceae [38254], Haptophyceae [2830], Rhodophyta [2763], Stramenopiles [33634]; ^3^Acanthamoebidae [33677], Alveolata [33630], Diplomonadida [207245], Entamoebidae [33084], Heterolobosea [5752], Jakobidae [143015], Mycetozoa [142796], Parabasalidea [5719]

### Full-length transcripts

The total number of clusters after EST clustering do not equal the number of protein encoding genes. This is mainly due to partial (as opposed to full-length) transcripts, i.e. a single gene is represented by more than one sequence because they do not overlap. How many of the clustered public (PPP) transcripts represent full-length coding sequences? Of those sequences that yield a filtered hit against plant mRNAs, 7.9% are putatively full-length. Of those, 53.9% start with Methionine, of the latter, 32.4% contain no X (X represents an indeterminable codon, which can be included by the ORF prediction).

### Orthologs, paralogs and mapping

**Figure 3:**
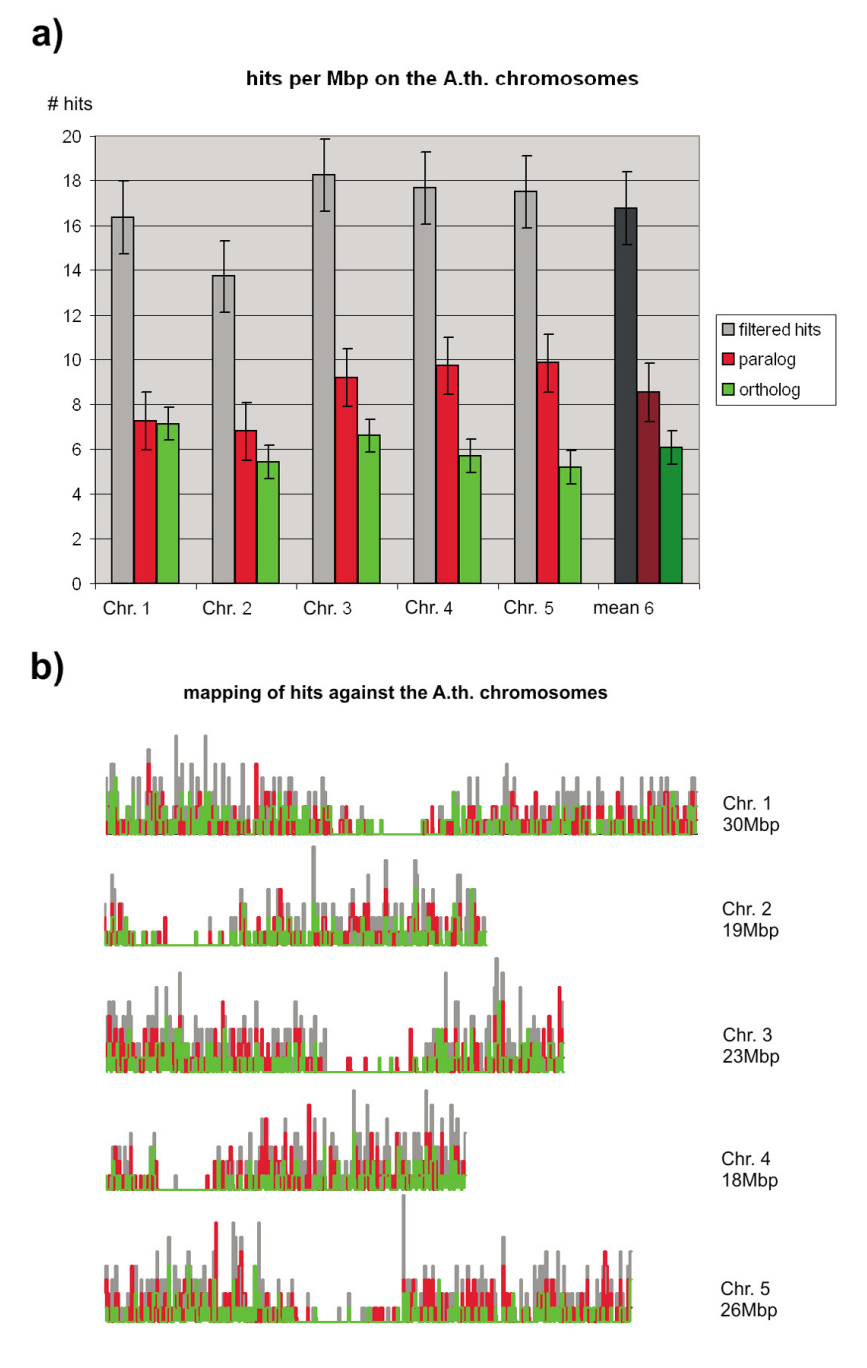
**Mapping of *Physcomitrella *transcripts to the *Arabidopsis *chromosomes**. Mapping of filtered BLAST hits (grey), paralogs (red) and orthologs (green) against the five *Arabidopsis *chromosomes (left to right / top to bottom). a) Hits per Mbp; error bars: average absolute deviation (AAD); column 6: mean values. b) Graphical representation using a finer granularity (100 kbp), each vertical step represents one hit.

The filtered hits against the *Arabidopsis *transcriptome (1,994 in total) were divided into non-redundant orthologs (722) and paralogs (1,015). As *Arabidopsis *orthologs, we defined all those sequences for which the initial subject matches the query in the reverse search (reciprocal hit). Paralogs were defined as those sequences for which this rule does not apply. This method of detecting potential orthologs has been used previously for cross-species comparisons (e.g. [17,18]). The three sequence sets were mapped against the *Arabidopsis *chromosomes using BLAST (Fig. 3). The distribution pattern clearly reveals the centromeric regions but otherwise does not display significant differences. Although there are some chromosome and sequence set-specific differences in the rate of hits per Mbp, these are not significant as measured by absolute average deviation.

### Taxonomic distribution and retained genes

The highest total number of non-redundant, filtered BLAST hits is (as expected) derived from the plant subset of the taxprot dataset (Table 1, Fig. 2a), followed by the animal and fungi subsets. When looking at the hits as percentage of the search space size (Fig. 2b), it becomes evident that quite a proportion of the sequence space of lower eukaryotes (including non-green algae) is covered. The comparatively high coverage of this "ancient" gene space suggests that mosses share many specialized genes with unicellular organisms.

**Figure 4:**
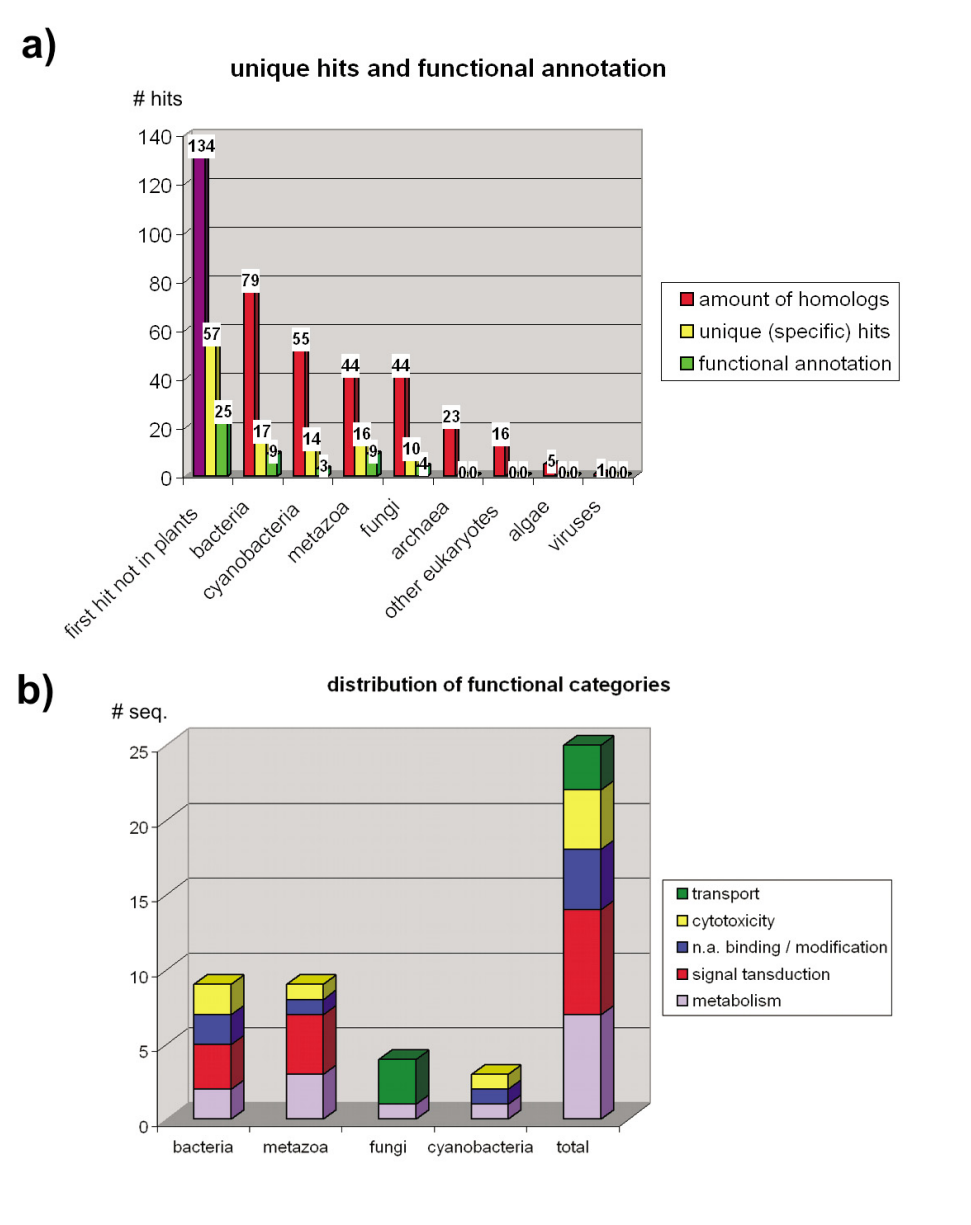
**Retained genes in moss: taxonomic distribution and functional categories**. a) *Physcomitrella *transcripts which have their best BLAST hit not among plants, divided by taxonomic category, further subdivided into specific hits (unique to a single taxonomic group – yellow) and those that could be assigned a putative function by means of homology searches (green). b) Distribution of functional categories among those taxonomic groups that yielded unique hits.

134 *Physcomitrella *ORF have their best BLAST hit not among plants (Fig. 4a). Consequently, these are candidates for horizontal gene transfer or, more likely, retained genes that were lost in seed plants during evolution. We had a closer look at those 57 transcripts which are specific to a single taxonomic group, namely bacteria, cyanobacteria, animals or fungi (unique hits). For 25 of those, a putative function could be assigned manually (Fig. 4b, Table 2). The broad functional categories of these taxon-specific retained genes are to some extent unevenly distributed. Whereas transport associated proteins are found solely among fungi, signal transduction gene products are found in both bacteria and animals. Transport and metabolism associated gene products support the wealth of secondary pathways found in moss (e.g., [14,15,19-22]), whereas the signal transduction genes also separate the moss from seed plants in this regard. Of special interest are two other functional categories among these candidate retained genes: cytotoxicity and nucleic acid modification. A broad range of cytotoxic abilities might explain why mosses can survive in moist environments mainly unplagued by microbial parasites, without the protection of a cuticula. Furthermore, it is, up until now, puzzling why *Physcomitrella *is able to integrate ectopic DNA into the genome by homologous recombination with an extraordinarily high rate [23,24] so far only found in bacteria and yeast, but in no other plant or any animal. Hints to unravel this mystery might be found in the presence of genes involved in DNA repair, binding and modification, as we discovered during this research.

**Table 2:** Functional annotation of retained genes into broad functional categories, assembled transcripts can be retrieved via .

**Pp transcript (putative retained gene)**	**taxonomic group**	**homolog**	**broad functional category (potential)**	**functional category**	**details**
PPP_2925_C1	bacteria	Membrane-bound lytic murein transglycosylase B	cytotoxicity	murein degradation	murein-degrading enzyme, may play a role in recycling of muropeptides during cell
BJ203770	bacteria	putative protease	cytotoxicity	protease	
PPP_4234_C1	metazoa	cytolysin I	cytotoxicity	cytotoxicity	involved in pore-formation
PPP_3510_C1	cyano	RTX toxins and related Ca2+-binding proteins	cytotoxicity	cytotoxicity	
PPP_1172_C1	bacteria	Enoyl-CoA hydratase/carnithine racemase	metabolism	fatty acid metabolism	
PPP_6629_C1	bacteria	mannosylglycerate synthase	metabolism	sugar metabolism	
PPP_5746_C1	metazoa	L-kynurenine 3-monooxygenase Fpk	metabolism	amino acid metabolism	
PPP_8479_C1	metazoa	COMMD2	metabolism	copper metabolism	COMM (copper metabolism MURR1) domain containing 2
BJ173412	metazoa	ubiquitin	metabolism	protein metabolism	ribosomal protein in C. elegans dehydrogenases
PPP_3987_C1	fungi	MNN9	metabolism	N-glycosylation	
PPP_6514_C1	cyano	oxidoreductase	metabolism	energy metabolism	related to aryl-alcohol dehydrogenases
PPP_11394_C1	bacteria	homolog of eukaryotic DNA ligase III	nucleic acid binding / modification	DNA repair	
BJ191550	bacteria	formamidopyrimidine-DNA glycosylase	nucleic acid binding / modification	DNA repair	
BJ160862	metazoa	Osa1 nuclear protein	nucleic acid binding / modification	DNA binding	chromatin regulation
BJ582496	cyano	SAM-dependent methyltransferase	nucleic acid binding / modification	nucleic acid modification	
PPP_2586_C1	bacteria	CarD protein	signal transduction	DNA binding	leucine zipper transcription factor, light- and starvation-induced response
PPP_3689_C1	bacteria	serine/threonine protein kinase	signal transduction	signal transduction	
BJ172132	bacteria	serine/threonine protein kinase	signal transduction	signal transduction	
PPP_460_C1	metazoa	HLA-B-associated transcript	signal transduction	signal transduction	
PPP_1041_C1	metazoa	calcium/calmodulin-dependent protein kinase II delta	signal transduction	signal transduction	
PPP_6326_C1	metazoa	tumor suppressor tout-velu	signal transduction	signal transduction	involved in diffusion of hedgehog
PPP_11399_C1	metazoa	dual-specificity tyrosine phosphatase YVH1	signal transduction	signal transduction	Non-receptor class dual specificity subfamily
PPP_184_C1	fungi	high-affinity iron permease	transport	transport	high affinity iron uptake
PPP_7115_C2	fungi	uric acid-xanthine permease	transport	transport	belongs to the Xanthine/Uracil oermeases family
PPP_11191_C1	fungi	inorganic phosphate transporter	transport	transport	probable inorganic phosphate transporter; yeast pho99 homologue

### Gene structure and splice sites

The average rate of introns per gene (~5) is the same in *Physcomitrella*, *Arabidopsis *and human [25]. The average *Physcomitrella *intron (252 bp) is longer than those of *Arabidopsis *(146 bp) and shorter than the typical human intron (740 bp). Furthermore, the *Physcomitrella *intron is longer than the exon, whereas in *Arabidopsis *it is the other way round. While the size distribution of *Arabidopsis *introns is centered around 70 bp, the longer moss introns are mainly clustered around 180 bp (data not shown). This fits the weak correlation of intron length and genome size generally found in eukaryotic organisms [26]. Intron positions of close homologs between *Physcomitrella *and *Arabidopsis *are generally conserved (e.g., [27]).

The *Physcomitrella *G/C content of 40% in the intron and 50% in the exon differs significantly from that of *Arabidopsis*; 33% and 44%, respectively. Generally, *Physcomitrella *introns contain more thymine (T) than the exons. In terms of mononucleotide composition, T is overrepresented in the intron and C is underrepresented in the exon. In terms of dinucleotides, there is a significant overrepresentation of TT in the introns. Outstanding trinucleotide usage are the overrepresented TTT in the intron and the stop codon TGA in the exon, while the other two stop codons TAA and TAG are underrepresented in the moss.

**Figure 5:**
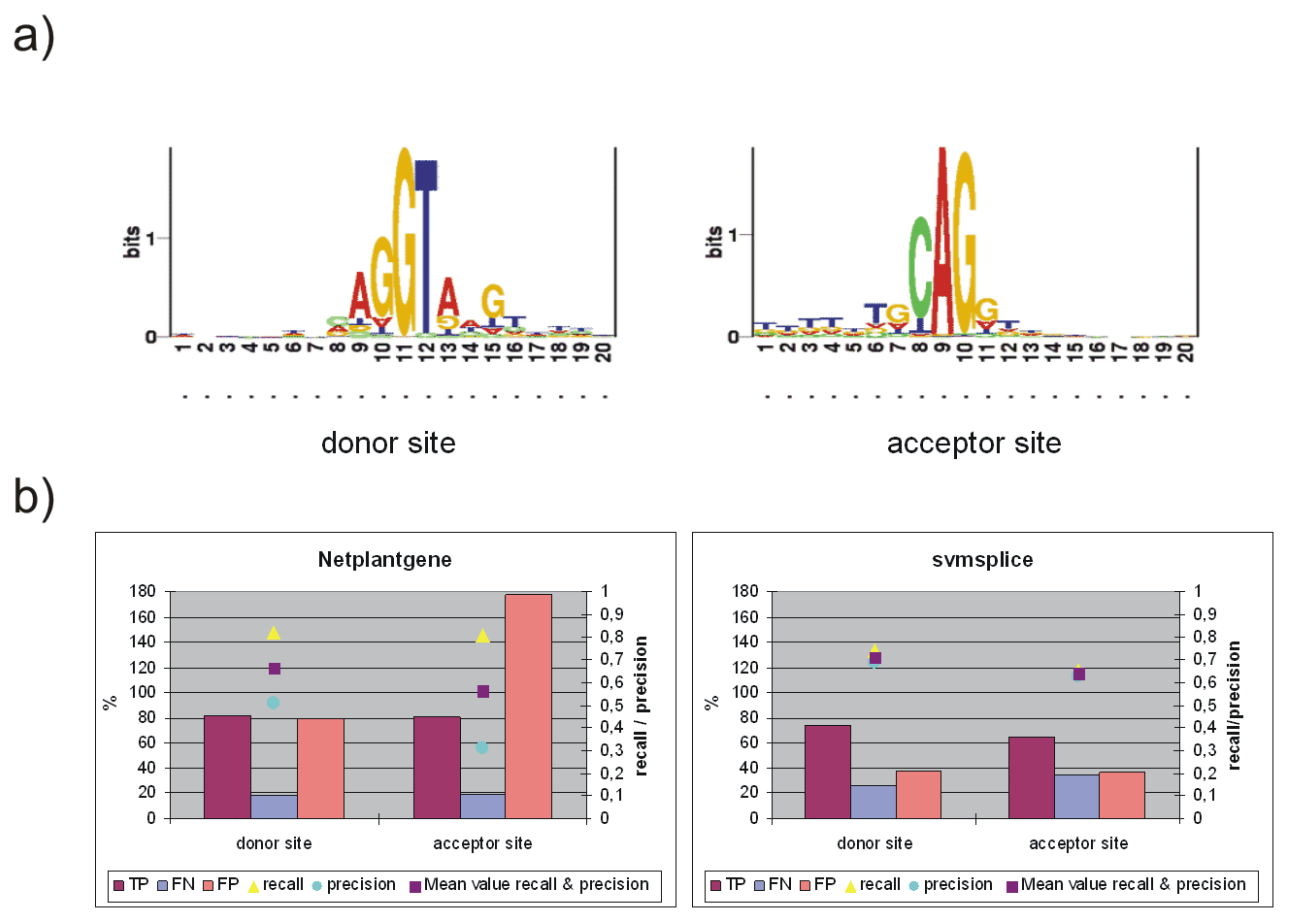
**Splice site sequence logos and efficiency of splice site prediction**. a) Sequence logos of *Physcomitrella *donor and acceptor sites. b) Prediction performance of Netplantgene and svmsplice for *Physcomitrella *splice sites. TP = true positive, FN = false negative, FP = false positive, measured on the lefthand (%) axis. Recall (sensitivity) = tp/(tp+fn), precision = tp/(tp+fp), measured on the righthand axis.

A visualisation of the *Physcomitrella *donor and acceptor sites is shown in figure 5a. Comparison of the *Arabidopsis*-trained Netplantgene [25] and the *Physcomitrella*-trained svmsplice (Fig. 5b) reveals a better overall performance of the support vector machine. Although Netplantgene exhibits a high recall, precision is low, which is due to the large amount of false positive predictions. Svmsplice predicts a lower rate of true positives (thus lower recall), however, precision is much better. The mean values of recall and precision for both donor and acceptor site are higher for svmsplice and thus make it the method of choice for accurate prediction of *Physcomitrella *splice sites.

### Composition of coding sequences and codon usage

**Figure 6:**
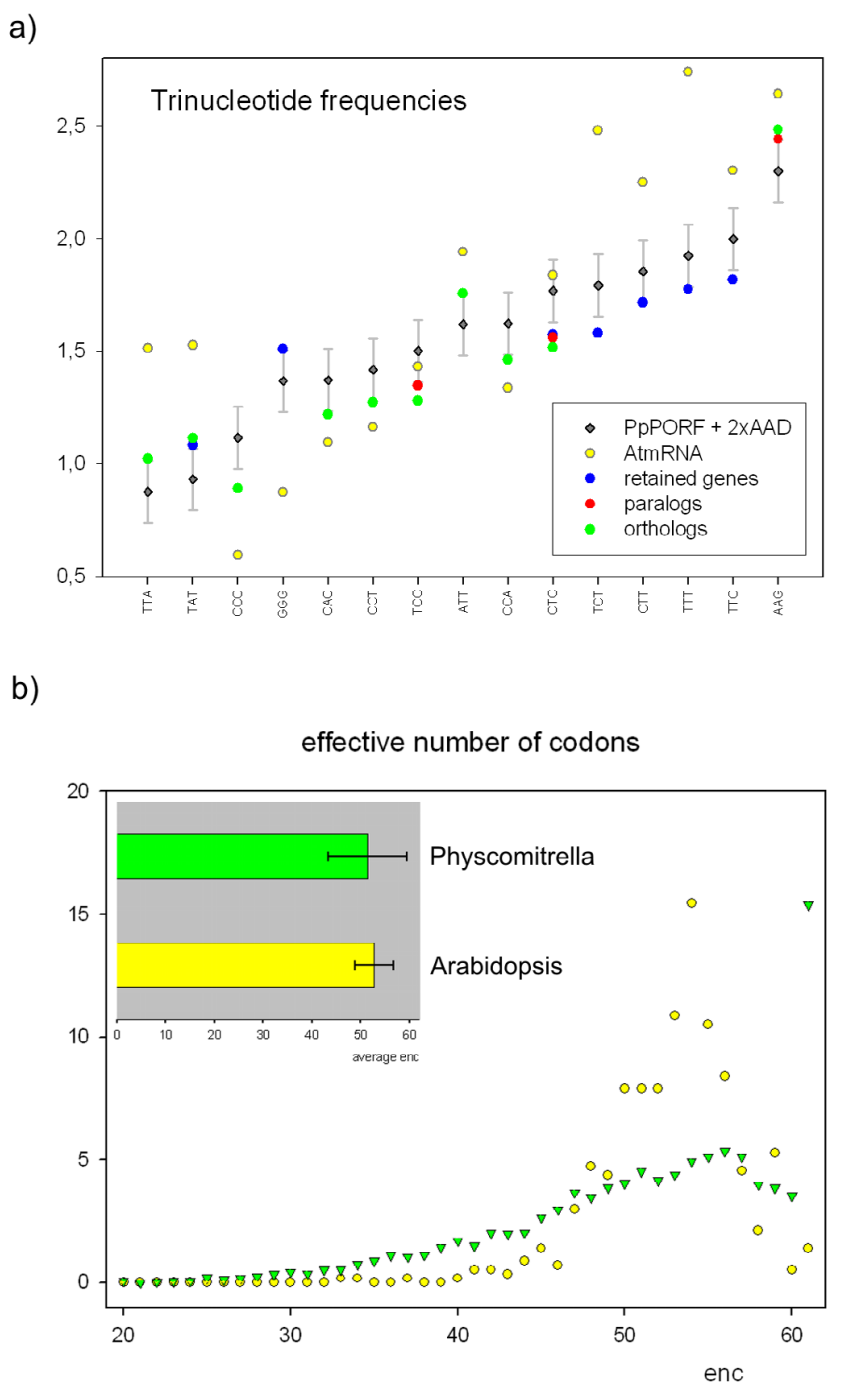
**Trinucleotide frequencies and codon usage**. a) The averaged *Physcomitrella *codon fraction usage measured as percentage of the total amount of counted codons is shown as grey diamonds, including a margin of 2× average absolute deviation (AAD, error bars), in comparison with *Arabidopsis *(yellow circles). Significantly deviating codons of the sequence subsets are presented as colored circles, namely retained genes (blue), paralogs (red) and orthologs (green). b) The effective number of codons (enc) for *Physcomitrella *(green) and *Arabidopsis *(yellow) as a range distribution scatter plot (y axis: % of analysed genes) and as averaged values (horizontal bar chart; error bars: standard deviation).

Significant differences in codon fraction usage for the three above mentioned sequence subsets (*Arabidopsis *orthologs and paralogs, retained genes) when compared with the averaged codon usage in *Physcomitrella *and *Arabidopsis *are shown in Fig. 6a. The Average G/C content of the *Arabidopsis *CDS is ~43%, whereas it is ~50% for *Physcomitrella *(Table 3). It might be argued that the EST-based estimation of G/C content in *Physcomitrella *is too high because of potential decay of AT-rich sequences [28]. However, when calculating the G/C content for all available 399 full-length CDS from Genbank, the percentage value is also ~50% (50.67%). This rate is also found in the retained genes and the *Arabidopsis *paralogs (Table 3), whereas the ortholog fraction has a significantly lower G/C content of ~49%, i.e. towards the *Arabidopsis *nucleotide composition. Codon bias in *Physcomitrella *is positively correlated with gene expression level and G/C content of the CDS [29]. It was argued that weak natural selection for translational efficiency is the driving force behind codon bias in the moss rather than mutational bias. Given the G/C rate of 50% in the CDS, a mutational bias indeed seems unlikely.

**Table 3:** Codon usage of *Physcomitrella *retained genes, orthologs and paralogs

**sequence set**	**# bases**	**mean # of each triplet**	**G/C content**	**# significant codon usage changes**	**codon usage towards At**	**codon usage away from At**	**codon over represented**	**codon under represented**	**significant changes per aa**
At mRNAs	10,755,859	56,020	43.32	n.a.	n.a.	n.a.	n.a.	n.a.	n.a.
Pp ORFs	7,638,122	39,782	49.94	n.a.	n.a.	n.a.	n.a.	n.a.	n.a.
retained genes	77,998	406	50.30	7	1	**6**	2	5	Phe under represented
paralogs	1,115,937	5,812	50.07	3	1	2	1	2	none
orthologs	953,293	4,965	**49.04**	10	**8**	2	4	6	Pro under reprensented
				sum	10	10	7	13	

The predicted *Physcomitrella *ORF were used as background to check for significant changes in percentage codon fraction usage in the orthologs, paralogs and retained genes (best BLAST hit not among plants). In case of significant deviation (two times average absolute deviation – AAD) from the total set, the direction of the change relative to the *Arabidopsis *codon usage was checked. Significant deviations are shown enlarged, At = *Arabidopsis thaliana*, Pp = *Physcomitrella patens*.

In retained genes, Phenylalanine codons are underrepresented, in the orthologs this is the case for Proline codons. As can also be seen from the G/C content drift mentioned above, the majority of deviating codons in the orthologs changed in the direction of the *Arabidopsis *percentage usage. For retained genes, it is the direct opposite: the significantly deviating codons in these genes point away from the *Arabidopsis *codon fraction usage. Orthologs are thought to be functionally equivalent across taxonomic groups. The common ancestor of land plants might have had a G/C content similar to mosses, i.e. around 50%. In order to preserve efficient functioning of orthologs it might have been necessary to evolve a slightly different codon usage for these genes in mosses, as is e.g. the case in *Arabidopsis*. The retained genes, on the other hand, are not found in seed plants and do not reflect the codon usage found there.

The average number of synonymous codons that is used in *Physcomitrella *and *Arabidopsis *CDS is not significantly different (Fig. 6b, bar chart). However, the percentage distribution of synonymous codon usage, as measured by the effective number of codons (enc), is surprisingly dissimilar (Fig. 6b, scatter plot). Most *Arabidopsis *coding sequences use a lot of synonymous codons (enc 45–59), whereas *Physcomitrella *displays a linear percentage increase from low to high values. Interestingly, around 15% of the moss genes contain no codon bias at all (enc = 61).

## Conclusion

The genome of the ancient land plant *Physcomitrella patens*, a moss, harbours genes of which at least 30% have a detectable homolog in seed plants. EST clustering yielded a database that covers a large proportion of the transcriptome, approximately 8% of the virtual transcripts contain full-length CDS.

Transcripts that are clear homologs of *Arabidopsis *genes were mapped against the *Arabidopsis *chromosomes, along with the set of paralogs and orthologs between the two organisms. All three sequence sets could be mapped evenly across the chromosomes, revealing neither hot nor cold spots (despite centromeric regions) nor differences in gene density.

While moss genes resemble those of *Arabidopsis*, there are significant differences. Introns are larger than those of the seed plant and are also longer than exons within moss, which is not the case in *Arabidopsis*. The G/C content of exons equals the A/T content. This might reflect a certain tenacity of the mosses' genetic system and its slow mutational rate. These might be necessary characteristics, as due to haploidy in the dominant gametophyte, the chance for the propagation of a disadvantageous change is higher than in a polyploid organism.

Whereas orthologs display a codon fraction usage drift towards *Arabidopsis*, the contrary is the case for retained genes. Thus, evolution of codon usage seems to be correlated with evolutionary history of protein genes. Mutational bias does not seem to play a role in the evolution of moss coding sequences. While the majority of *Physcomitrella *CDS displays codon bias, there is a significant fraction (~15%) of genes that is not biased at all, possibly representing a more ancient nucleotide composition than oberved in *Arabidopsis*. Splice sites in the moss resemble those in *Arabidopsis*, however, species-specific prediction models, like the one presented here, are necessary in order to avoid false positives. The same is true for the prediction of ORF based on EST data.

A high proportion of the sequence space of unicellular eukaryotes is covered by moss homologs, which apparently have not been lost since the days of the last common ancestor. The majority of moss genes find their best scoring homolog in plants. However, there are 134 putative retained genes that have their best BLAST hit among other taxonomic groups. Of those, 57 genes are specific to a single taxonomic group, putative functional annotation could be carried out for 25 of these proteins. The functional annotation revealed deviations in the taxonomic distributions: certain sets of genes seem to be shared with specific taxonomic groups, for example, transport proteins with fungi or signal transduction genes with bacteria and animals. Of special interest are genes that are possibly involved in cytotoxicity, metabolism and nucleic acid repair. These genes might be the reasons for some of the extraordinary capabilities of mosses, namely resistance against microbial pathogens, additional secondary pathways (as compared with seed plants) and a high rate of homologous recombination.

## Methods

### Clustering of EST data

All publicly available protein encoding DNA sequences of *Physcomitrella *were retrieved using Entrez [30] and divided into 399 "seeds" (full length mRNA sequences) as well as 102,535 EST and other sequences. This dataset is called the *Physcomitrella patens *public set, or PPP.

A set of 17 moss-specific repetitive elements, detected mainly in the untranslated regions of *Physcomitrella *genes [31] and used for filtering (see below) is available via cosmoss.org [32]. Filtering, clustering and assembly of EST data were done using the Paracel transcript assembler, PTA [33]. A species-specific parameter set has been developed and is available upon request.

For sequences where electropherograms were available, base-calling was carried out using phred [34]. Base quality values of EST sequences without available sequencer raw data was set arbitrarily to a low value, 10%, and in the case of seed sequences to a higher confidence value of 50%. Filtering included steps for removal of synthetic (vector/linker) and low quality sequences as well as of contaminants (homologs of *E. coli *as well as *Physcomitrella *mitochondrial, rRNA and chloroplast genes). Low-complexity regions were annotated together with poly-A tails, untranslated regions (UTR, UTRdb see [35]) and repetitive elements (repeats, repbase see [36]), in order not to disturb clustering and assembly. In a final step, sequences containing less than 150 bases of sense characters were removed. For PPP, a total of 100,079 sequences went into the clustering.

Prior to clustering, homologs of the seed sequences were pulled out of the sequence pool and assembled independently. Where possible, sequences were placed into 5' and 3' partitions based on detected poly-A tails and inherent annotated information. Both during clustering and assembly, putative chimeras (cloning artefacts) were detected and tagged. During assembly, contigs were built within clusters and putative splice variants detected. After clustering and assembly, the PPP set contained a total of 26,131 sequences. By using only the longest sequence in each cluster, a non-redundant set of 22,218 sequences was produced. The PP dataset contained 63,685 sequences in the complete and 48,961 sequences in the non-redundant set.

### Splice site prediction

For the splice site prediction, all publicly available pairs of genomic and cDNA/mRNA sequences were retrieved (40 genes). Together with 29 unpublished sequences, these sequences were aligned using MGAlign 1.3.6 [37] in order to determine the splice sites. The procedure yielded a total of 438 exons and 368 introns. The complete dataset is available via cosmoss.org [32].

The sequence logos (Fig. 5a) were created via the web interface at [38] using 10 nucleotides up- and downstream of the donor / acceptor sites.

Suppor vector machine: The software used for training and classification was SVMlight [39], libsvm [40] and svmsplice [41]. The complete set of splice sites was divided into training/testing sets of sizes 10–90%, for each set three samples were drawn. The set containing 90% of the sites for training proved to yield the best results. Optimization of parameters was done by 10-fold cross-validation, plotting precision vs. recall and chosing the best curve. The best performing model could be constructed using 50 nucleotides up- and downstream of the splice sites as context with the basepairing feature set of svmsplice and a polynomial kernel function of 4^th ^order.

### BLAST searches and filtering

BLAST searches were carried out using Paracel BLAST [33], a parallelized version of BLAST 2 [42], on amino acid level whenever applicable. In order to exclude random hits which are not based on true sequence homology, alignments had to contain at least 30% identical positions and a minimum length of 100 amino acid characters. This rigorous filtering excludes some true positive hits but removes almost all false positives [43]. Putative full-length CDS had to pass the same filtering. In addition, in this case only those hits were counted that covered at least 90% of the subjects length. For the determination of identical sequences, BLASTN was performed and hits were filtered to be at least 95% identical and 300 nucleotides long. Non-redundant hits were counted by removing all subjects that were present more than once in the search result.

### Additional sequence datasets

The predicted coding sequences of rice (56,056 sequences) and *Arabidopsis *(28,581 sequences) genes were taken from release 1.0 and 4.0 of the TIGR database [44], respectively. The taxprot dataset (3,346,100 sequences, see Table 1 for details) was created by downloading the respective sequences from Genbank [30] using appropriate Entrez queries. All three datasets consist of amino acid sequences. The *Arabidopsis thaliana *chromosome sequences were retrieved from Genbank [45].

### ORF prediction

ESTScan 2.0 [46] was used to predict open reading frames. The species-specific model for *Physcomitrella *was built by using the 399 public full length seed sequences (complete mRNAs) mentioned above. ORF were predicted from the clustered EST data (non-redundant datasets). For the PPP set, 19,081 ORF were predicted; 34,981 for the PP set. Predictions were done using both the *Arabidopsis *and the *Physcomitrella *model for comparison. Manual inspection of several known CDS revealed that the *Arabidopsis*-based prediction contained false-positive stretches, which was not the case for the *Physcomitrella*-based prediction. Although the *Physcomitrella *model predicted a lower number of ORF, it was used in order to keep false-positives to a minimum.

### Codon usage

Four different sets of coding sequences were used (see table 3). A set of 7,765 well annotated *Arabidopsis *mRNAs was retrieved using Entrez. The *Physcomitrella *datasets contained the predicted ORF for the complete PPP set (19,081 sequences), the *Arabidopsis *paralogs (1,659) and orthologs (1,476) described above as well as the putatively retained genes not found in higher plants (134). The smallest set contained 77,998 nucleotides and thus a theoretical average of 406 instances of each triplet, which allows significant analyses.

Nucleotide frequencies were calculated with the GCG 10.3 [47] software composition. Codon usage fractions for individual datasets were calculated as percentage of the respective total amount of counted codons. Absolute deviations in comparison to the full *Physcomitrella *ORF set were calculated for the three subsets (retained genes, *Arabidopsis *orthologs and paralogs). The computed mean value over all sets (average absolute deviation) was 0.069. Codon fraction usage deviation was counted as significant only if it differed at least twice as much (+/- 0.138%) from the full set.

The effective number of codons (enc) was calculated using CodonW (J. Peden, [48]). The enc values range from 20 (maximum bias, i.e. only one synonymous codon is used per amino acid) to 61 (no bias, all synonymous codons are being used).

## Abbreviations

CDS = coding sequence(s), EST = expressed sequence tag(s), ORF = open reading frame(s)

## Authors' contributions

SAR carried out most of the analyses, drafted the manuscript and designed the work. DF carried out the splice site prediction. DL participated in the analyses and generated the databases and the web interface. RR participated in drafting and conception of the manuscript. All authors read and approved the final manuscript.
